# Interventions targeting non-symptomatic cases can be important to prevent local outbreaks: SARS-CoV-2 as a case study

**DOI:** 10.1098/rsif.2020.1014

**Published:** 2021-05-19

**Authors:** Francesca A. Lovell-Read, Sebastian Funk, Uri Obolski, Christl A. Donnelly, Robin N. Thompson

**Affiliations:** ^1^ Mathematical Institute, University of Oxford, Oxford, UK; ^2^ Department of Statistics, University of Oxford, Oxford, UK; ^3^ Centre for Mathematical Modelling of Infectious Diseases, London School of Hygiene and Tropical Medicine, London, UK; ^4^ Porter School of the Environment and Earth Sciences, Tel Aviv University, Tel Aviv, Israel; ^5^ School of Public Health, Tel Aviv University, Tel Aviv, Israel; ^6^ MRC Centre for Global Infectious Disease Analysis, Department of Infectious Disease Epidemiology, Imperial College London, London, UK; ^7^ Mathematics Institute, University of Warwick, Coventry, UK; ^8^ The Zeeman Institute for Systems Biology and Infectious Disease Epidemiology Research, University of Warwick, Coventry, UK

**Keywords:** mathematical modelling, infectious disease epidemiology, SARS-CoV-2, COVID-19, presymptomatic infection, asymptomatic infection

## Abstract

During infectious disease epidemics, an important question is whether cases travelling to new locations will trigger local outbreaks. The risk of this occurring depends on the transmissibility of the pathogen, the susceptibility of the host population and, crucially, the effectiveness of surveillance in detecting cases and preventing onward spread. For many pathogens, transmission from pre-symptomatic and/or asymptomatic (together referred to as non-symptomatic) infectious hosts can occur, making effective surveillance challenging. Here, by using SARS-CoV-2 as a case study, we show how the risk of local outbreaks can be assessed when non-symptomatic transmission can occur. We construct a branching process model that includes non-symptomatic transmission and explore the effects of interventions targeting non-symptomatic or symptomatic hosts when surveillance resources are limited. We consider whether the greatest reductions in local outbreak risks are achieved by increasing surveillance and control targeting non-symptomatic or symptomatic cases, or a combination of both. We find that seeking to increase surveillance of symptomatic hosts alone is typically not the optimal strategy for reducing outbreak risks. Adopting a strategy that combines an enhancement of surveillance of symptomatic cases with efforts to find and isolate non-symptomatic infected hosts leads to the largest reduction in the probability that imported cases will initiate a local outbreak.

## 1. Introduction

Emerging epidemics represent a substantial challenge to human health worldwide [1–4]. When cases are clustered in specific locations, two key questions arise: (i) will exported cases lead to local outbreaks in new locations? and (ii) which surveillance and control strategies in those new locations will reduce the risk of local outbreaks?

Branching process models are used for a range of diseases to assess whether cases that are newly arrived in a host population will generate a local outbreak driven by sustained local transmission [5–11]. These models can also be used to predict the effectiveness of potential control interventions. For example, early in the coronavirus disease 2019 (COVID-19) pandemic, Hellewell *et al.* [12] used simulations of a branching process model to predict whether new outbreaks would fade out under different contact tracing strategies. Thompson [13] estimated the probability of local outbreaks analytically using a branching process model and found that effective isolation of infectious hosts leads to a substantial reduction in the outbreak risk.

A factor that can hinder control interventions during any epidemic is the potential for individuals to transmit a pathogen while not showing symptoms. For COVID-19, the incubation period has been estimated to last approximately 5 or 6 days on average [14,15], and pre-symptomatic transmission can occur during that period [16–20]. In addition, asymptomatic infected individuals (those who never develop symptoms) also contribute to transmission [16,21,22].

Motivated by the need to assess the risk of outbreaks outside China early in the COVID-19 pandemic, we show how the risk that imported cases will lead to local outbreaks can be estimated using a branching process model. Unlike standard approaches for estimating the probability of a major epidemic analytically [23–26], non-symptomatic individuals are included in the model explicitly. By using a function that characterizes the efficacy of interventions for different surveillance efforts (denoted *f*(*ρ*, *δ*) in the model), we explore the effects of interventions that aim to reduce this risk. Under the assumption that detected infected hosts are isolated effectively, we consider whether it is most effective to dedicate resources to enhancing surveillance targeting symptomatic individuals, to instead focus on increasing surveillance for non-symptomatic individuals or to use a combination of these approaches.

We show that, when surveillance resources are limited, the maximum reduction in the outbreak risk almost always corresponds to a mixed strategy involving enhanced surveillance of both symptomatic and non-symptomatic hosts. This remains the case even if the surveillance effort required to find non-symptomatic infected individuals is substantially larger than the effort required to find symptomatic individuals. This highlights the benefits of not only seeking to find and isolate symptomatic hosts but also dedicating resources to detecting non-symptomatic cases during infectious disease epidemics.

## 2. Methods

### 2.1. Model

We consider a branching process model in which infectious individuals are classified as asymptomatic (*A*), pre-symptomatic (*I*_1_) or symptomatic (*I*_2_). Hosts in any of these classes may generate new infections. The parameter *ξ* represents the proportion of new infections that are asymptomatic, so that a new infection either involves increasing *A* by one (with probability *ξ*) or increasing *I*_1_ by one (with probability 1 − *ξ*).

Pre-symptomatic hosts may go on to develop symptoms (transition from *I*_1_ to *I*_2_) or be detected and isolated (so that *I*_1_ decreases by one). Symptomatic individuals (*I*_2_) can be isolated (so that *I*_2_ decreases by one) or be removed due to recovery or death (so that again *I*_2_ decreases by one). Similarly, asymptomatic hosts may be detected and isolated or recover (so that *A* decreases by one in either case).

**Figure 1.**
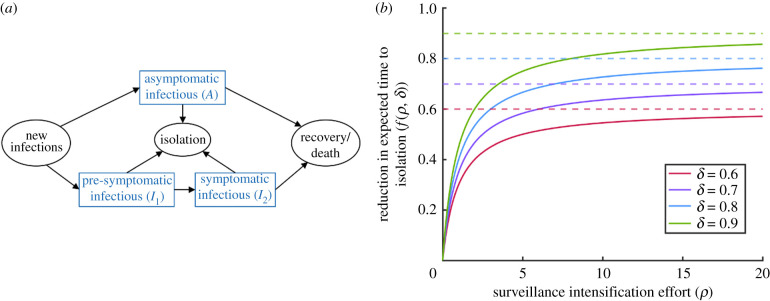
The branching process model used in our analyses. (*a*) Schematic showing the different event types in the branching process model. The parameters of the model are described in the text and in table 1. (*b*) The relationship between the surveillance intensification effort (*ρ*) and the proportional reduction in the expected time to isolation (*f*(*ρ*, *δ*)), shown for different values of the parameter *δ* (solid lines). The parameter *δ* ∈ (0, 1) represents the upper bound of *f*(*ρ*, *δ*) (dotted lines). This general functional relationship between surveillance effort and isolation effectiveness is assumed to hold for surveillance of both non-symptomatic and symptomatic individuals, although non-symptomatic hosts are more challenging to detect than symptomatic hosts (*ɛ* < 1).

A schematic showing the different possible events in the model is shown in figure 1*a*. The analogous compartmental differential equation model to the branching process model that we consider is given by

$$\begin{array}{rl} \frac{dA}{dt} & =\xi (\eta \beta A+\alpha \beta I_{1}+\beta I_{2})-\frac{\varepsilon \gamma }{1-f(\rho_{1},\delta )}A-\nu A,\\ \frac{dI_{1}}{dt} & =(1-\xi )(\eta \beta A+\alpha \beta I_{1}+\beta I_{2})-\frac{\varepsilon \gamma }{1-f(\rho_{1},\delta )}I_{1}-\lambda I_{1}\\ \mathrm{and}\;\frac{dI_{2}}{dt} & =\lambda I_{1}-\frac{\gamma }{1-f(\rho_{2},\delta )}I_{2}-\mu I_{2}. \end{array}$$

The parameters of the model, and the form of the function *f*(*ρ*, *δ*) that describes how the expected time to isolation is reduced for a given surveillance effort, are outlined below.

In our model, the parameter *β* and its scaled counterparts *αβ* and *ηβ* represent the rates at which symptomatic, pre-symptomatic, and asymptomatic hosts generate new infections, respectively. Since we are modelling the beginning of a potential local outbreak, we assume that the size of the susceptible population remains approximately constant and do not track the depletion of this population. The parameter *λ* governs the rate at which pre-symptomatic individuals develop symptoms, so that the expected duration of the pre-symptomatic period is 1/*λ* days in the absence of interventions. Similarly, without interventions, the expected durations of the symptomatic and asymptomatic infectious periods are 1/*μ* days and 1/*ν* days, respectively.

The baseline rate at which symptomatic individuals are detected and isolated is determined by the parameter *γ*. Assuming that non-symptomatic individuals are more difficult to detect than symptomatic individuals, we take the analogous quantity for non-symptomatic hosts to be *ɛγ*, where the scaling factor *ɛ* < 1 reflects the fact that interventions targeting non-symptomatic hosts are likely to be less effective for the same surveillance effort. We assume that the sensitivity of surveillance is identical for pre-symptomatic and asymptomatic individuals and therefore use the same isolation rate for both of these groups.

The parameters *ρ*_1_ and *ρ*_2_ represent the surveillance intensification effort targeted at non-symptomatic and symptomatic hosts, respectively. The function f(ρ, δ)=δρ/(1+ρ) governs the proportional reduction in the expected time to isolation for a given surveillance effort, *ρ* (for a similar approach in which the proportion of infectious cases prevented is assumed to be a function of control effort, see Matthews *et al.* [27]). The functional form of *f*(*ρ*, *δ*) is chosen for three main reasons. First, it generates a reduced expected time to isolation when the surveillance effort increases. Second, since the proportional reduction in the expected time to isolation is bounded above by the parameter *δ* ∈ (0, 1), the isolation rate saturates and cannot increase indefinitely. Third, the gradient ∂f/∂ρ decreases with the surveillance effort *ρ*, meaning that an increase in the surveillance effort has a greater impact at low surveillance efforts compared to when this effort is already large [27]. The function *f*(*ρ*, *δ*) is shown in figure 1*b* for different values of the parameter *δ*.

### 2.2. Reproduction number

The basic reproduction number, *R*_0_, represents the expected number of secondary infections generated by a single infected individual introduced at the start of their infection into a fully susceptible population in the absence of intensified surveillance:

$$R_{0}=\frac{\xi \eta \beta }{\nu +\epsilon \gamma }+(1-\xi )\left[\frac{\alpha \beta }{\lambda +\epsilon \gamma }+\frac{\lambda }{\lambda +\epsilon \gamma }\;\frac{\beta }{\gamma +\mu }\right].$$

This expression is the sum of the expected number of transmissions from a host who begins in the asymptomatic class and from a host who begins in the pre-symptomatic infectious class, weighted by the respective probabilities *ξ* and 1 − *ξ* that determine the chance that the host experiences a fully asymptomatic course of infection. The expected number of transmissions from a host who begins in the pre-symptomatic infectious class comprises transmissions occurring during the incubation period and transmissions occurring during the symptomatic period, accounting for the possibility that the host is isolated before developing symptoms.

The proportion of infections arising from pre-symptomatic hosts in the absence of intensified surveillance is then given by

2.1 $$K_{p}=\frac{\left(1-\xi )\alpha /(\lambda +\epsilon \gamma \right)}{\xi \eta /(\nu +\epsilon \gamma )+(1-\xi )[\alpha +\lambda /(\gamma +\mu )]/(\lambda +\epsilon \gamma )},$$

and the equivalent quantity for asymptomatic hosts is given by

2.2 $$K_{a}=\frac{\xi \eta /(\nu +\epsilon \gamma )}{\xi \eta /(\nu +\epsilon \gamma )+(1-\xi )[\alpha +\lambda /(\gamma +\mu )]/(\lambda +\epsilon \gamma )}.$$

### 2.3. Baseline values of model parameters

Since this study was motivated by the need to estimate outbreak risks outside China in the initial stages of the COVID-19 pandemic, we used a baseline set of parameter values in our analyses that was informed by studies conducted during this pandemic (table 1). Where possible, these parameter values were obtained from the existing literature. However, we also performed sensitivity analyses to determine how our results varied when the parameter values were changed (see electronic supplementary material, text S3 and figures S3–S12). In table 1, and throughout, rounded values are given to three significant figures.

**Table 1.** Parameters of the model and the values used in the baseline version of our analysis.

parameter	meaning	baseline value	justification
*R*_0_	expected number of secondary infections caused by a single infected individual (when *ρ*_1_ = *ρ*_2_ = 0)	*R*_0_ = 3	within estimated range for SARS-CoV-2 [28–31]
*ξ*	proportion of infections that are asymptomatic	*ξ* = 0.2	[32–34]
*β*	rate at which symptomatic individuals generate new infections	*β* = 0.336 days^−1^	chosen so that *R*_0_ = 3
*α*	relative infectiousness of pre-symptomatic individuals compared to symptomatic individuals	*α* = 2.78	chosen so that 48.9% of transmissions arise from pre-symptomatic hosts (i.e. *K_p_* = 0.489) [16]
*η*	relative infectiousness of asymptomatic individuals compared to symptomatic individuals	*η* = 0.519	chosen so that 10.6% of transmissions arise from asymptomatic hosts (i.e. *K_a_* = 0.106) [16]
*γ*	isolation rate of symptomatic individuals without intensified surveillance	*γ* = 0.0924 days^−1^	chosen so that 1/(γ+μ)=4.6 days [35]
*ɛ*	relative isolation rate of non-symptomatic individuals without intensified surveillance (compared to symptomatic individuals)	*ɛ* = 0.1	assumed; chosen within the range *ɛ* ∈ (0, 1) (for different values, see electronic supplementary material, figure S7)
*λ*	rate at which pre-symptomatic individuals develop symptoms	*λ* = 0.5 days^−1^	[20]
*μ*	recovery rate of symptomatic individuals	*μ* = 1/8 days^−1^	[36–38]
*ν*	recovery rate of asymptomatic individuals	*ν* = 0.1 days^−1^	chosen so that, in the absence of interventions, the expected duration of infection is identical for all infected hosts (1/*ν* = 1/*λ* + 1/*μ*)
*δ*	upper bound on the fractional reduction in the time to isolation	*δ* = 0.8	assumed; chosen within the natural range *δ* ∈ (0, 1) (for different values, see electronic supplementary material, figure S11)
*ρ*_1_	surveillance intensification effort targeted at non-symptomatic hosts	*ρ*_1_ allowed to vary in the range [0, 20]	N/A—range of values explored
*ρ*_2_	surveillance intensification effort targeted at symptomatic hosts	*ρ*_2_ allowed to vary in the range [0, 20]	N/A—range of values explored

The value of the parameter governing the baseline rate at which symptomatic individuals are isolated, *γ*, was chosen to match empirical observations, which indicate that individuals who seek medical care before recovery or death do so around 4–6 days after symptom onset [35]. Specifically, we assumed that the period of time to the first medical visit could be used a proxy for the time to isolation, and chose *γ* so that the expected time period to isolation conditional on isolation occurring during the symptomatic period was given by 1/(γ+μ)=4.6 days [35]. This is different to the time period that we refer to as the expected time to isolation for symptomatic hosts, which is 1/*γ* days (see Methods).

### 2.4. Probability of a local outbreak

For stochastic simulations of compartmental epidemiological models starting from a small number of hosts infected initially, there are generally two qualitatively different types of behaviours. The pathogen may fade out rapidly, or case numbers may begin to increase exponentially (only starting to fade out once the number of susceptible individuals has been sufficiently depleted, unless public health measures are introduced to reduce transmission). Consequently, running many simulations of those types of model with *R*_0_ larger than but not close to one, the epidemic size is distributed bimodally, with the total number of individuals ever infected falling into one of two distinct ranges (for a simple example, see electronic supplementary material figure S1A; see also refs. [39–41]). In that scenario, a natural definition for the probability of a local outbreak is therefore the proportion of outbreak simulations for which the total number of infected individuals falls into the higher of these two ranges.

Here, since we are considering the initial phase of potential local outbreaks, we instead considered a branching process model in which depletion of susceptibles was not accounted for. If simulations of branching process models are run, then in each simulation, the pathogen either fades out with few infections or case numbers generally increase indefinitely. The probability of a local outbreak starting from a small number of infected hosts then corresponds to the proportion of simulations in which the pathogen does not fade out quickly and case numbers increase indefinitely instead. This again provides a natural definition of a local outbreak since simulations can be partitioned into two distinct sets (for an example in which simulations of a simple branching process model are used to calculate the probability of a local outbreak, see electronic supplementary material, figure S1B).

As an alternative to repeated simulation, we instead use our branching process model (figure 1*a*) to perform analytic calculations of the probability that a single imported infectious host initiates a local outbreak. To do this, we denote the probability of a local outbreak not occurring, starting from *i* pre-symptomatic hosts, *j* symptomatic hosts, and *k* asymptomatic hosts, by *q_i_*_,*j*,*k*_. Starting from one pre-symptomatic host (so that *i* = 1 and *j* = *k* = 0), there are four possibilities for the next event. That host could:

(i) generate a new asymptomatic infection (with probability *ξαβ*/[*αβ* + *λ* + ɛ*γ*/(1 − *f*(*ρ*_1_, *δ*))]);
(ii) generate a new pre-symptomatic infection (with probability (1 − *ξ*)*αβ*/[*αβ* + *λ* + ɛ*γ*/(1 − *f*(*ρ*_1_, *δ*))]);
(iii) develop symptoms (with probability *λ*/[*αβ* + *λ* + *ɛγ*/(1 − *f*(*ρ*_1_, *δ*))]); or
(iv) be isolated (with probability [*ɛγ*/(1 − *f*(*ρ*_1_, *δ*))]/[*αβ* + *λ* + *ɛγ*/(1 − *f*(*ρ*_1_, *δ*))]).

These probabilities are obtained by considering the rates at which different possible events occur in the branching process model. Pre-symptomatic hosts generate new infections at rate *αβ*, and these new infections occur in asymptomatic and pre-symptomatic hosts with probabilities *ξ* and 1 − *ξ*, respectively. Therefore, starting from a single pre-symptomatic host, new asymptomatic infections occur at rate *ξαβ*, while new pre-symptomatic infections occur at rate (1 − *ξ*)*αβ*. In addition, pre-symptomatic hosts develop symptoms at rate *λ*, and are isolated at rate $\epsilon \gamma /(1-f(\rho_{1},\delta ))$. The overall rate at which events occur is the sum of these individual event rates:

$$\mathrm{total}\;\mathrm{event}\;\mathrm{rate}=\alpha \beta +\lambda +\frac{\;\epsilon \gamma }{1-f(\rho_{1},\delta )}.$$

For each of the four possible next events ((i)–(iv), earlier), the probability that event occurs next is the individual rate at which that event occurs divided by the total event rate, leading to the expressions given.

We use these probabilities to condition on the event that occurs next in the branching process, following the introduction of a single pre-symptomatic infectious individual into the population. If that event is the generation of a new asymptomatic infection, which occurs with probability $\xi \alpha \beta /(\alpha \beta +\lambda +\varepsilon \gamma /(1-f(\rho_{1},\delta )))$, the probability that a local outbreak subsequently does not occur is *q*_1,0,1_. Applying analogous reasoning to the other possible events, we obtain

$$\begin{array}{rl} q_{1,0,0}=\; & \frac{\xi \alpha \beta }{\alpha \beta +\lambda +\varepsilon \gamma /(1-f(\rho_{1},\delta ))}q_{1,0,1}\\ & +\frac{(1-\xi )\alpha \beta }{\alpha \beta +\lambda +\varepsilon \gamma /(1-f(\rho_{1},\delta ))}q_{2,0,0}\\ & +\frac{\lambda }{\alpha \beta +\lambda +\varepsilon \gamma /(1-f(\rho_{1},\delta ))}q_{0,1,0}\\ & +\frac{\varepsilon \gamma /(1-f(\rho_{1},\delta ))}{\alpha \beta +\lambda +\varepsilon \gamma /(1-f(\rho_{1},\delta ))}q_{0,0,0}. \end{array}$$

If there are no infectious hosts present in the population (i.e. *i* = *j* = *k* = 0), then a local outbreak will not occur and so *q*_0,0,0_ = 1. Assuming that transmission chains arising from two infectious individuals are independent gives *q*_1,0,1_ = *q*_1,0,0_ *q*_0,0,1_ and $q_{2,0,0}=q_{1,0,0}^{2}$. Hence,

2.3 $$q_{1,0,0}=a\xi q_{1,0,0\;}q_{0,0,1}+a(1-\xi )q_{1,0,0}^{2}+bq_{0,1,0}+(1-a-b),$$

where $a=\alpha \beta /(\alpha \beta +\lambda +\varepsilon \gamma /(1-f(\rho_{1},\delta )))$ and b=λ/(αβ+λ+ $\varepsilon \gamma /(1-f(\rho_{1},\delta )))$.

Similarly, considering the probability of a local outbreak failing to occur starting from a single symptomatic host gives

$$\begin{array}{rl} q_{0,1,0}=\; & \frac{\xi \beta }{\beta +\gamma /(1-f(\rho_{2},\delta ))+\mu }q_{0,1,1}+\frac{(1-\xi )\beta }{\beta +\gamma /(1-f(\rho_{2},\delta ))+\mu }q_{1,1,0}\\ & +\frac{\gamma /(1-f(\rho_{2},\delta ))+\mu }{\beta +\gamma /(1-f(\rho_{2},\delta ))+\mu }q_{0,0,0}. \end{array}$$

As before, noting that *q*_0,0,0_ = 1 and assuming that different infection lineages are independent leads to

2.4 $$q_{0,1,0}=c\xi q_{01,0}q_{0,0,1}+c(1-\xi )q_{1,0,0}q_{0,1,0}+(1-c),$$

where $c=\beta /(\beta +\gamma /(1-f(\rho_{2},\delta ))+\mu )$.

Finally, considering the probability of a local outbreak failing to occur starting from a single asymptomatic host gives

2.5 $$q_{0,0,1}=d\xi q_{0,0,1}^{2}+d(1-\xi )q_{1,0,0}q_{0,0,1}+(1-d),$$

where $d=\eta \beta /(\eta \beta +\nu +\varepsilon \gamma /(1-f(\rho_{1},\delta )))$.

Equations (2.3), (2.4) and (2.5) may be combined to give a single quartic equation for *q*_0,0,1_, yielding four sets of solutions for *q*_1,0,0_, *q*_0,1,0_ and *q*_0,0,1_ (see electronic supplementary material, text S1). It is straightforward to verify that *q*_1,0,0_ = *q*_0,1,0_ = *q*_0,0,1_ = 1 is always a solution, and further solutions can be found numerically. The appropriate solution to take is the minimal non-negative real solution $q_{1,0,0}=q_{1,0,0}^{*},\;q_{0,1,0}=q_{0,1,0}^{*},\;q_{0,0,1}=q_{0,0,1}^{*}$ (see electronic supplementary material, text S1). Then, the probability of a local outbreak occurring beginning from a single pre-symptomatic host is given by

$$p_{1,0,0}=1-q_{1,0,0}^{*},$$

with equivalent expressions holding for *p*_0,1,0_ and *p*_0,0,1_ (the probability of a local outbreak occurring beginning from a single symptomatic host or a single asymptomatic host, respectively).

Throughout, we consider the probability *p* of a local outbreak starting from a single non-symptomatic host entering the population, accounting for the possibility that the non-symptomatic host is either pre-symptomatic or asymptomatic:

$$p=(1-\xi )p_{1,0,0}+\xi p_{0,0,1}.$$

## 3. Results

### 3.1. Probability of a local outbreak

**Figure 2.**
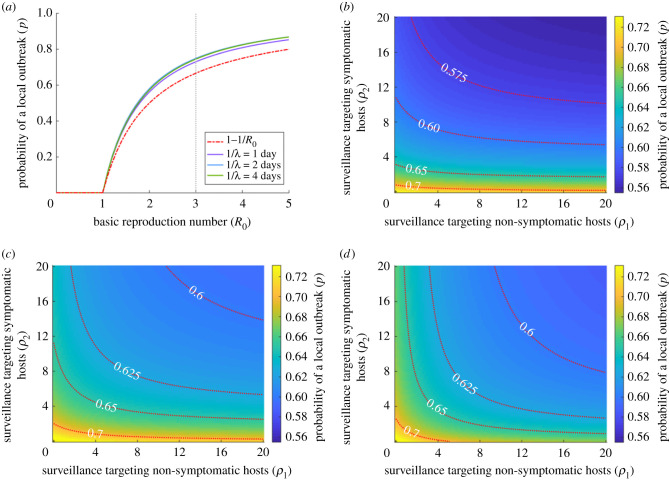
The effect of the duration of the pre-symptomatic and asymptomatic periods on the probability of a local outbreak (*p*), starting from a single non-symptomatic host. (*a*) The probability of a local outbreak as a function of the basic reproduction number *R*_0_, for pre-symptomatic periods of lengths 1/*λ* = 1 day (purple), 1/*λ* = 2 days (blue) and 1/*λ* = 4 days (green) in the absence of enhanced surveillance (*ρ*_1_ = *ρ*_2_ = 0). In each case, the duration of the asymptomatic period (1/*ν*) is adjusted so that the relative proportion of infections arising from asymptomatic hosts compared to pre-symptomatic hosts remains constant (*K_a_*/*K_p_* = 0.218, as in the baseline case). The red dash-dotted line indicates the probability of a local outbreak in the absence of non-symptomatic transmission. The vertical grey dotted line indicates *R*_0_ = 3, the baseline value used throughout. (*b*) The probability of a local outbreak as a function of the surveillance intensification efforts *ρ*_1_ and *ρ*_2_, for 1/*λ* = 1 day. (*c*) The analogous figure to B but with 1/*λ* = 2 days. (*d*) The analogous figure to B but with 1/*λ* = 4 days. Red dotted lines indicate contours of constant local outbreak probability (i.e. lines on which the probability of a local outbreak takes the values shown). The value of *β* is varied in each panel to fix *R*_0_ = 3. All other parameter values are held fixed at the values in table 1 (except where stated).

We considered the effect of *R*_0_ and the duration of the pre-symptomatic and asymptomatic periods on the probability of a local outbreak when a non-symptomatic host enters a new host population (figure 2). We examined pre-symptomatic periods of length 1/*λ* = 1 day, 1/*λ* = 2 days and 1/*λ* = 4 days; in each case, the duration of the asymptomatic period (1/*ν* days) was adjusted so that the relative proportion of infections arising from asymptomatic hosts compared to pre-symptomatic hosts remained fixed (*K_a_*/*K_p_* = 0.218, as in the baseline case). If instead non-symptomatic infections are not accounted for, the infectious period follows an exponential distribution and the probability of a local outbreak is given by *p* = 1 − 1/*R*_0_ (red dash-dotted line in figure 2*a*). Including non-symptomatic infection in the model therefore led to an increased risk of a local outbreak in the absence of surveillance intensification (figure 2*a*).

We then considered the dependence of the probability of a local outbreak on the intensity of surveillance targeting non-symptomatic and symptomatic hosts (figure 2*b–d*). The maximum value of the surveillance intensification effort that we considered (given by *ρ*_1_ or *ρ*_2_ values of 20) corresponded to a 76% reduction in the expected time to isolation (blue line in figure 1*b*), i.e. a 76% reduction in 1/*ɛγ* or 1/*γ*.

**Figure 3.**
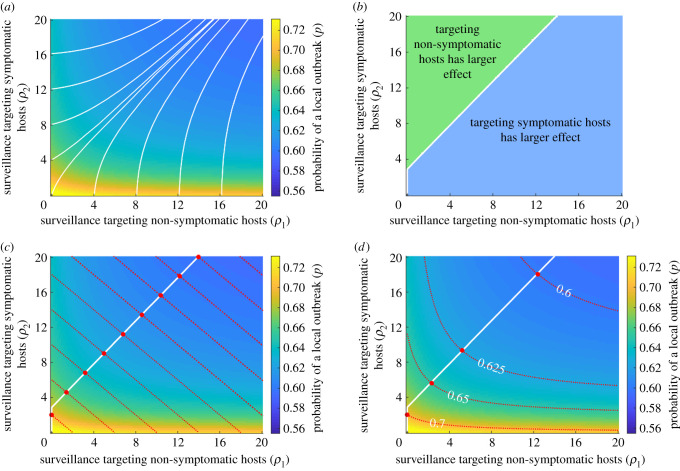
Optimal surveillance strategies to reduce the probability of a local outbreak (*p*) starting from a single non-symptomatic host. (*a*) The local outbreak probability for different values of *ρ*_1_ and *ρ*_2_, with the steepest descent contours overlaid (white lines). For the maximum reduction in the probability of a local outbreak at each point, surveillance must be enhanced for both non-symptomatic and symptomatic individuals, with different levels of prioritization depending on the current values of *ρ*_1_ and *ρ*_2_. (*b*) Values of *ρ*_1_ and *ρ*_2_ for which increasing surveillance for non-symptomatic hosts (i.e. increasing *ρ*_1_) is more effective at reducing the local outbreak probability than increasing surveillance for symptomatic hosts (i.e. increasing *ρ*_2_) (green region) and vice versa (blue region). The white line represents the steepest descent contour starting from *ρ*_1_ = *ρ*_2_ = 0, under the constraint that surveillance can only be enhanced for either symptomatic or non-symptomatic hosts at any time. The diagonal section of the steepest descent contour is made up of small horizontal and vertical sections. (*c*) Strategies for minimizing the local outbreak probability for a given fixed total surveillance effort (*ρ*_1_ + *ρ*_2_ = *C*). Red dotted lines indicate contours on which *ρ*_1_ + *ρ*_2_ is constant, and red circles indicate the points along these contours at which the local outbreak probability is minimized. The white line indicates the optimal surveillance enhancement strategy if the maximum possible surveillance level (i.e. the maximum value of *ρ*_1_ + *ρ*_2_ = *C*) is increased. (*d*) Strategies for minimizing the surveillance effort required to achieve a pre-specified risk level (an ‘acceptable’ local outbreak probability). Red dotted lines indicate contours of constant local outbreak probability (i.e. lines on which the probability of a local outbreak takes the values shown); red circles indicate the points along these contours at which the total surveillance effort *ρ*_1_ + *ρ*_2_ is minimized. The white line indicates the optimal strategy to follow if the pre-specified risk level is increased or reduced.

The length of the pre-symptomatic and asymptomatic periods significantly affected the dependence of the probability of a local outbreak on the level of surveillance targeted at non-symptomatic and symptomatic hosts. In figure 2*b*, in which the duration of the pre-symptomatic period was 1 day, increasing surveillance targeted at non-symptomatic hosts (*ρ*_1_) had a limited effect on the probability of a local outbreak, while increasing surveillance targeted at symptomatic hosts (*ρ*_2_) had a more significant effect. For example, increasing the surveillance effort targeted at non-symptomatic hosts to *ρ*_1_ = 5 (a 67% reduction in the time to isolation) only reduced the probability of a local outbreak from 0.730 to 0.716, whereas the equivalent effort targeted at symptomatic hosts (*ρ*_2_ = 5) reduced the probability to 0.630. As shown in figure 3*c,d*, however, when the pre-symptomatic and asymptomatic periods were longer, the benefit of directing surveillance resources towards detecting non-symptomatic individuals increased. This was because longer pre-symptomatic and asymptomatic periods increased the proportion of infections generated by non-symptomatic individuals (*K_p_* + *K_a_*, see eqns (2.1) and (2.2)); a pre-symptomatic period of 1 day, 2 days and 4 days corresponded to values of *K_p_* + *K_a_* equal to 0.424, 0.595 and 0.746, respectively.

### 3.2. Optimizing surveillance enhancement

We next considered in more detail the impact of surveillance targeted at non-symptomatic hosts (*ρ*_1_) relative to the impact of surveillance targeted at symptomatic hosts (*ρ*_2_). For our baseline parameter values, we considered the probability of a local outbreak starting from a single imported non-symptomatic individual for a range of values of *ρ*_1_ and *ρ*_2_. We calculated the steepest descent contours (white lines in figure 3*a*) numerically using a gradient maximization approach, in which at each point the contour direction was determined by minimizing the local outbreak probability over a fixed search radius (see electronic supplementary material, text S2 and figure S2). These contours indicate how *ρ*_1_ and *ρ*_2_ should be altered to maximize the reduction in the probability of a local outbreak. In this case, enhancing surveillance targeting both symptomatic and non-symptomatic hosts is always optimal (the steepest descent contours are neither horizontal nor vertical).

We then considered a scenario in which, at any time, it is only possible to direct resources towards enhancing surveillance of either non-symptomatic individuals or symptomatic individuals (e.g. antigen testing of non-symptomatic contacts of known infectious individuals, or screening for symptomatic individuals at public events). In figure 3*b*, the blue region represents values of *ρ*_1_ and *ρ*_2_ for which enhancing surveillance targeting symptomatic hosts (i.e. increasing *ρ*_2_) leads to a larger reduction in the local outbreak probability than enhancing surveillance targeting non-symptomatic hosts (i.e. increasing *ρ*_1_). In contrast, in the green region, enhancing surveillance of non-symptomatic individuals is more effective than enhancing surveillance of symptomatic individuals. The white line represents the steepest descent contour starting from *ρ*_1_ = *ρ*_2_ = 0, under the constraint that surveillance can be enhanced only for symptomatic or non-symptomatic hosts at any time.

Practical deployment of surveillance is often subject to logistical constraints, and policy makers may wish to design surveillance strategies to achieve a specific objective—for example, to maximize the effectiveness of limited resources or to minimize the cost of achieving a desired outcome. We therefore also considered the following two examples of such objectives.

#### 3.2.1. Objective 1: minimize the probability of a local outbreak for a fixed total surveillance effort

First, we considered the question: given a fixed maximum surveillance effort (*ρ*_1_ + *ρ*_2_ = *C*), how should surveillance be targeted at non-symptomatic and symptomatic hosts? This involves setting the values of *ρ*_1_ and *ρ*_2_ to minimize the local outbreak probability. The optimal strategies in this case are shown in figure 3*c*. The red dotted lines represent contours along which the total surveillance effort *ρ*_1_ + *ρ*_2_ is held constant (i.e. different values of *C*). On each contour, the red circle indicates the point at which the local outbreak probability is minimized.

If surveillance resources are increased (i.e. *C* increases), a further question is how surveillance should then be increased. In figure 3*c*, the white line represents the contour of steepest descent, under the constraint that the total change in surveillance effort (*ρ*_1_ + *ρ*_2_) is held constant at each step (rather than a constant search radius, as shown in figure 3*a*—for more details, see electronic supplementary material, text S2 and figure S2). This contour coincides exactly with that shown in figure 3*b*.

These results indicate that if surveillance resources are such that *C* is greater than 2.8 (corresponding to a 59% reduction in the time to isolation of symptomatic hosts), the optimal surveillance strategy involves both enhanced surveillance of symptomatic individuals and non-symptomatic individuals (the red dots correspond to strictly positive values of both *ρ*_1_ and *ρ*_2_, unless *C* is less than 2.8).

#### 3.2.2. Objective 2: minimize the total surveillance effort to achieve a pre-specified reduction in the probability of a local outbreak

Second, we considered the question: given a pre-specified acceptable risk level (i.e. probability of a local outbreak), how should the surveillance level targeted at non-symptomatic and symptomatic hosts be chosen? This involves choosing *ρ*_1_ and *ρ*_2_ to minimize *ρ*_1_ + *ρ*_2_ along a given contour corresponding to a fixed local outbreak probability (red dotted lines in figure 3*d*). On each contour, the red circle indicates the point along that contour at which the total surveillance effort *ρ*_1_ + *ρ*_2_ is minimized. These optimal points also lie exactly along the line on which enhancing surveillance targeted at symptomatic hosts is equally effective compared to enhancing surveillance targeted at non-symptomatic hosts.

As long as the target local outbreak probability is less than 0.69, optimal surveillance involves enhanced surveillance of non-symptomatic individuals as well as symptomatic individuals. For example, to reduce the local outbreak probability to 0.6, the optimal approach is to deploy resources such that *ρ*_1_ = 12.4 (a 74% reduction in the time to isolation of non-symptomatic individuals) and *ρ*_2_ = 18.0 (a 76% reduction in the time to isolation of symptomatic individuals).

Plots analogous to figure 3*d* in which the parameters were varied from their baseline values are shown in electronic supplementary material, figures S3–S12. In each case that we considered, our main finding remained unchanged. There always exists a threshold local outbreak probability such that, if the target local outbreak probability is below this threshold, the optimal strategy for further reduction in the local outbreak probability involves enhancing surveillance targeting both non-symptomatic and symptomatic individuals.

## 4. Discussion

A key component of infectious disease epidemic management is inferring the risk of outbreaks in different locations [5–8,11,41,42]. Surveillance and control strategies can be introduced to reduce the risk that imported cases will lead to local outbreaks [12,13,43–46]. However, for a range of pathogens, public health measures are hindered by non-symptomatic infectious hosts who can transmit the pathogen yet are challenging to detect [16,42,44,47–49].

Here, we showed how the probability of a local outbreak can be estimated using a branching process model that accounts for transmission from non-symptomatic infected individuals (figure 1). The model can be used to assess the local outbreak probability for different surveillance strategies that target non-symptomatic or symptomatic hosts (figure 2). Previous studies have shown that detection of non-symptomatic infections can be a key component of epidemic forecasting [42] and containment [44] and have demonstrated the benefits of identifying and isolating infectious non-symptomatic hosts to reduce transmission [16,17]. We focused instead on investigating how surveillance should be targeted at non-symptomatic or symptomatic hosts to reduce the probability that cases imported to new locations will trigger a local outbreak (figure 3*a,b*). We also showed how the optimal surveillance level targeting these two groups can be assessed when surveillance resources are limited and policy makers have specific objectives (figure 3*c*,*d*). In each case, our main conclusion was that surveillance for non-symptomatic infected hosts (*ρ*_1_ > 0) can be an important component of reducing the local outbreak risk during epidemics. This result has broad implications, and our analysis could be extended to assess the potential for containing outbreaks at their source using a range of specific interventions targeting symptomatic and non-symptomatic hosts.

Our goal here was to use the simplest possible model to explore the effects of surveillance of non-symptomatic and symptomatic individuals on the risk of local outbreaks. However, this model is not without its limitations. One area of uncertainty is the precise values of the parameters governing pathogen transmission and control. In this article, we chose a baseline set of parameter values that is consistent with the findings of studies conducted during the COVID-19 pandemic, although constructing a detailed transmission model for this pandemic was not our main focus. For example, we set the relative rates at which pre-symptomatic and asymptomatic individuals generate new infections compared to symptomatic individuals so that 48.9% of transmissions arise from pre-symptomatic infectors and 10.6% arise from asymptomatic infectors [16]. While this is in line with reported estimates [50,51], there is substantial variation between studies. Similarly, the proportion of individuals who experience a fully asymptomatic course of infection (denoted by *ξ* in our model) is subject to a considerable degree of uncertainty. Here, we chose *ξ* = 0.2 as the baseline value [32–34], but estimates in the literature range from 0.04 to over 0.8 [33,52–54]. We therefore also conducted sensitivity analyses in which we explored a range of different values of model parameters (electronic supplementary material, text S3 and figures S3–S12). In each case that we considered, our main conclusion was unchanged: surveillance of non-symptomatic individuals can contribute to reducing the risk of local outbreaks. This result is expected to hold for epidemics of any pathogen for which non-symptomatic individuals contribute significantly to transmission.

For our modelling approach to be used to make precise quantitative predictions during epidemics, it would be necessary to update the model to include the range of different specific surveillance and control interventions that are in place. For example, detection of non-symptomatic infected individuals is facilitated by contact tracing and antigen testing, which are carried out routinely during epidemics and can be included in models explicitly [12,44,55,56]. Reductions in contacts due to social distancing strategies and school or workplace closures could also be accounted for [57,58], although such interventions are often introduced after a local outbreak has begun rather than in the initial phase of a potential local outbreak as considered here. We modelled the level of surveillance targeted at non-symptomatic and symptomatic hosts in a simple way using a function describing the relationship between surveillance effort and effectiveness (figure 1*b*). We assumed that this general functional relationship could be applied to interventions targeting both symptomatic and non-symptomatic hosts, accounting for logistical differences in the ease of targeting either group by scaling the effectiveness of surveillance for non-symptomatic hosts using the parameter *ɛ* (results are shown for different values of *ɛ* in electronic supplementary material, figure S8). In principle, it would be possible to include entirely different functional forms describing the relationship between surveillance effort and effectiveness for strategies targeting symptomatic and non-symptomatic individuals, and these could be tailored to the effects of particular interventions. If different public health measures are included in the model explicitly, then it would be possible to increase the accuracy of assessments of the relative public health benefits of specific interventions that only target symptomatic individuals (e.g. screening for passengers with heightened temperatures at airports [59,60]) compared to interventions that also target non-symptomatic hosts (e.g. travel bans or quarantine of all inbound passengers [61,62]). Of course, this would require data from which the relative effectiveness of different measures could be inferred.

The underlying transmission model could also be extended to include additional realism in several ways. Transmission dynamics are influenced by marked heterogeneities in the patterns of contacts between individuals in different age groups [63,64], and, for SARS-CoV-2, susceptibility to infection, the likelihood of developing symptoms, and the average severity of those symptoms increase with age [65,66]. Age-dependent variation in the proportion of asymptomatic cases in particular implies that the optimal balance of surveillance between symptomatic and non-symptomatic hosts may differ between age groups. An age-structured version of the model presented here is a focus of our ongoing research. Similarly, for a range of infectious diseases, the distribution characterizing the number of secondary infections generated by each infected host (the offspring distribution) exhibits a high degree of overdispersion [67–70]. For a fixed value of *R*_0_, a higher degree of overdispersion increases the likelihood that initial cases will fade out without leading to a local outbreak [71,72] and suggests that greater reductions in local outbreak risks could theoretically be achieved for the same surveillance effort if potential superspreaders or superspreading events can be identified and targeted.

Despite the necessary simplifications, we have shown how the risk of local outbreaks can be estimated during epidemics using a branching process model that includes non-symptomatic infectious hosts explicitly. Determining the extent to which non-symptomatic individuals contribute to transmission is essential early in emerging epidemics of a novel pathogen. As we have shown, if transmissions occur from non-symptomatic infectors, dedicating surveillance resources towards finding non-symptomatic cases can be an important component of public health measures that aim to prevent local outbreaks.

## Data accessibility

The computer code used to perform the analyses in this article is available as part of the supplementary material [73]. All computer code was written in the MATLAB programming environment (version R2019a).
